# ChatGPT passing USMLE shines a spotlight on the flaws of medical education

**DOI:** 10.1371/journal.pdig.0000205

**Published:** 2023-02-09

**Authors:** Amarachi B. Mbakwe, Ismini Lourentzou, Leo Anthony Celi, Oren J. Mechanic, Alon Dagan

**Affiliations:** 1 Department of Computer Science, Virginia Tech, Blacksburg, Virginia, United States of America; 2 Institute for Medical Engineering and Science, Massachusetts Institute of Technology, Cambridge, Massachusetts, United States of America; 3 Department of Medicine, Beth Israel Deaconess Medical Center, Beth Israel Deaconess Medical Center, Boston, Massachusetts, United States of America; 4 Department of Biostatistics, Harvard T.H. Chan School of Public Health, Boston, Massachusetts, United States of America; 5 eMed Digital Healthcare, Miami, Florida, United States of America; 6 Department of Emergency Medicine, Mount Sinai Medical Center, Miami Beach, Florida, United States of America; 7 Department of Emergency Medicine and Critical Care, Herbert Wertheim College of Medicine at Florida International University, Miami, Florida, United States of America; 8 Department of Emergency Medicine, Beth Israel Deaconess Medical Center, Boston, Massachusetts, United States of America; University of Pittsburgh, UNITED STATES

Artificial Intelligence (AI) has recently spurred revolutionary innovations in digital health, with large language models (LLMs) making significant contributions. LLMs are deep neural network models with large parameter spaces. These models, containing billions of parameters, are typically trained on gigabytes or even terabytes of text data. LLMs represent an important advance in AI, enabling new possibilities for natural language understanding and generation.

Since the public release of ChatGPT in November of 2022, the conversation around AI and its role in society has reached a tipping point. For the first time, a sophisticated LLM has become accessible to the broad public in an exceptionally easy to access format. Initial reactions were nothing short of amazement, with an explosion across public media lauding the algorithm as having the potential to “change our mind about how we work, how we think, and what human creativity really is".

Over the subsequent weeks, the LLM employed was tested with challenges of increasing complexity, often utilizing standardized examinations as a way to determine how the algorithm stacks up against the humans for whom that the tests were designed. Despite its lack of domain specific training, ChatGPT has not disappointed, often passing or near-passing examinations designed for post-graduate levels of specialization across a broad range of fields [1]. In this issue, Kung et al. report the performance of ChatGPT on the United States Medical Licensing Examinations (USMLE). What does this kind of performance mean for us? [2]

As we learn more about its capabilities, we must consider what ChatGPT’s success on a medical examination says about testing and current medical education. The goal of the USMLE exam is to assess “a physician’s ability to apply knowledge, concepts, and principles, and to demonstrate fundamental patient-centered skills that are important in health and disease and that constitute the basis of safe and effective patient care” [3]. While achieving these goals does require some rote memorization, we are increasingly realizing that the ability to regurgitate mechanistic models of health and disease, as in what often takes place during rounds, may be less important in this age of information being rapidly accessible at your fingertips. We have also come to appreciate that there are other important characteristics, such as problem-solving ability, familiarity with information resources, a strong work ethic, respect for patients and the entire care team, courtesy, warmth, and humility that make one an excellent clinician beyond having an appropriate breadth of medical knowledge. Hence, exams like the USMLE fail to fully assess the skills required for modern medical practice.

ChatGPT’s success also reflects the rigidity in the way medicine is taught, wherein there is a right and wrong answer (that an AI chatbot could pick out), while the ‘right’ answer may be far more nuanced and contextually dependent. The framing of medical knowledge as something that can be encapsulated into multiple choice questions creates a cognitive framing of false certainty. Medical knowledge is often taught as fixed model representations of health and disease. Treatment effects are presented as stable over time despite constantly changing practice patterns. Mechanistic models are passed on from teachers to students with little emphasis on how robustly those models were derived, the uncertainties that persist around them, and how they must be recalibrated to reflect advances worthy of incorporation into practice.

Equally frightening, is the observation that potentially biased internet medical content (which ChatGPT is trained on) is sufficient to pass a medical examination. These biases stem from research performed in high-income countries and textbooks that describe studies of patients which may not be representative of the global population [4]. These fragilities are exacerbated by sampling selection with content from high-powered academic institutions dominating the science of health and disease. Current medical education does not assess students’ ability to identify or mention potential biases in their knowledge: in general, it does not even make students aware of these issues.

The direct application of AI to standard test taking runs the risk of replicating biases in the data on which these models are trained. There is little mention of the horror stories from proprietary algorithms that were deployed without robust evaluation for errors and bias [5–7]. AI is notoriously bad at nuance and context, when there are no clear rules, and when experts can disagree about the right answer. And if there’s a body of knowledge that requires nuance and context, prescribes guidelines even when the evidence is less than solid, and is maintained by experts who often disagree about the right answer, it will be medicine. Those at the table with the loudest voice, which in this case produces the content that dominates the internet, will shape the input, and therefore the output of LLMs.

Learning is about leveraging the current body of knowledge, understanding its gaps, and seeking to fill those gaps. It requires being comfortable with and being able to probe the uncertainties. We fail as teachers by not teaching students how to understand the gaps in the current body of knowledge. We fail them when we preach certainty over curiosity, and hubris over humility. Medical education also requires being aware of the biases in the way medical knowledge is created and validated. These biases are best addressed by optimizing the cognitive diversity within the community. More than ever, there is a need to inspire cross-disciplinary collaborative learning and problem-solving. Medical students need data science skills that will allow every clinician to contribute to, continually assess, and recalibrate medical knowledge.

ChatGPT lacks thoughtful reasoning like humans, and its passing score emphasizes that the current version of USMLE is mostly focused on the rote memorization of mechanistic models of health and disease. But this is far from the actual practice of medicine that is predicated on human interactions, and for these reasons, AI will never replace the nurses, the doctors and other professionals at the frontlines of care. Undoubtedly, AI and LLMs will transform every facet of what we do, from research and writing to graphic design and medical diagnosis. However, its current success in passing standardized test after standardized test is an indictment of *what* and *how* we train our doctors, our lawyers, and our students in general.

ChatGPT passed an examination that rewards memorizing the components of a system rather than analyzing how it works, how it fails, how it was created, how it is maintained. Its success demonstrates some of the shortcomings in how we train and evaluate medical students. Critical thinking requires appreciation that ground truths in medicine continually shift, and more importantly, an understanding how and why they shift. Perhaps the most important lesson from the success of LLMs in passing examinations such as the USMLE is that now is the time to rethink how we train and evaluate our students. The glory in medicine has traditionally gone to the innovators, and they are certainly critically important. But just as important are those clinicians who fully leverage the knowledge, skills, and treatments we already possess, and have the time, willingness, and ability to pass these on to, and create more relevant and valid assessments for, the next medical generation.

## References

[1] Varanasi Lakshmi. ChatGPT is on its way to becoming a virtual doctor, lawyer, and business analyst. Here’s a list of advanced exams the AI bot has passed so far. Businessinsider. 2023. Available from: https://www.businessinsider.com/list-here-are-the-exams-chatgpt-has-passed-so-far-2023-1

[2] Kung TH, Cheatham M, Medenilla A, Sillos C, De Leon L, Elepaño C, et al. (2023) Performance of ChatGPT on USMLE: Potential for AI-assisted medical education using large language models. PLOS Digit Health 2(2): e0000198. doi: 10.1371/journal.pdig.0000198 PMC9931230 36812645

[3] United States Medical Licensing Examination (USMLE). Available from: https://www.usmle.org/

[4] Data shows racial disparities in Alzheimer’s disease diagnosis between black and white research study participants. National institute of health 2021. Available from: https://www.nia.nih.gov/news/data-shows-racial-disparities-alzheimers-disease-diagnosis-between-black-and-white-research

[5] Chicago pd predictive policing heat list. THE VERGE 2021. Available from: https://www.theverge.com/c/22444020/chicago-pd-predictive-policing-heat-list

[6] Hill Kashmir. A Dad took photos of his naked toddler for the doctor. Google flagged him as a criminal. The New York Times. 2022. Available from: https://www.nytimes.com/2022/08/21/technology/google-surveillance-toddler-photo.html

[7] Researchers find AI is bad at predicting GPA, grit, eviction, job training, layoffs, and material hardship. VentureBeat 2020. Available from: https://venturebeat.com/ai/ai-is-bad-at-predicting-gpa-grit-eviction-job-training-layoffs-and-material-hardship /

